# *Hericium erinaceus* Extracts Exert Gastroprotective Effects Through Modulation of the Gut Microbiota and Metabolites

**DOI:** 10.1002/fsn3.71851

**Published:** 2026-05-11

**Authors:** Ruixin Bei, Lifan Yu, Zhonghua Lu, Tingxuan Zong, Yanfang Sun

**Affiliations:** ^1^ College of Life Sciences and Medicine Zhejiang Sci‐Tech University Hangzhou China; ^2^ Zhejiang International Joint Laboratory of Traditional Medicine and Big Health Products Development Hangzhou China; ^3^ Zhejiang Provincial Agricultural Technology Extension Center Hangzhou China

**Keywords:** acute gastric ulcer, gut microbiota, *H. erinaceus* extract (HEE), metabolomics, proteomics

## Abstract

*Hericium erinaceus* ( *H. erinaceus* ) is a traditional edible and medicinal fungus with a long history of use in gastrointestinal protection. Although its therapeutic potential in gastric health has been widely acknowledged, the underlying mechanisms by which it modulates the gut microbiota and affects metabolic profiles in the context of gastric diseases remain incompletely understood. On the basis of the “gut microbiota and metabolites” regulatory network, this study elucidated the mechanism of *H. erinaceus* extract (HEE) in alleviating ethanol‐induced acute gastric injury. The results showed that HEE alleviated ethanol‐induced acute gastric injury, downregulated 12 (S)‐HETE, and enriched short‐chain fatty acid (SCFA)‐producing bacteria. Notably, the water extract of *H. erinaceus* (WEHE) additionally elevated propionic acid. These SCFA‐producing bacteria were positively correlated with propionic acid but negatively correlated with 12 (S)‐HETE. These results suggest that WEHE may enhance the protective effect on the stomach by regulating the “gut microbiota‐metabolites” axis.

## Graphical abstract

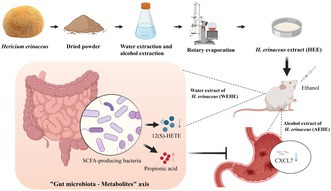

## 1 Introduction

Gastric ulcer is a common type of peptic ulcer disease. In 2019, an estimated 8.09 million individuals worldwide were affected by peptic ulcers, marking a 25.82% rise over the preceding three decades (Xie et al. 2022). Peptic ulcer disease represents a growing global health burden. Multiple factors such as *Helicobacter pylori* infection, alcohol consumption, and long‐term use of non‐steroidal anti‐inflammatory drugs can all contribute to the formation of Gastric ulcer (Stermer 2002; Lanas and Chan 2017; Gupta et al. 2023). Among these, excessive alcohol intake markedly elevates the risk of mucosal erosion and gastrointestinal hemorrhage. In view of the widespread abuse of alcohol, peptic ulcer caused by alcohol has become a common gastrointestinal disease (Zhou et al. 2021). Furthermore, a substantial body of research has demonstrated that the homeostasis of the gut microbiota is closely associated with gastric health. Various gastric disorders, including chronic gastritis, gastric ulcer, and gastric cancer, are frequently accompanied by alterations and dysbiosis in the composition of the intestinal microbiota (Huang et al. 2021; Karakan et al. 2021). The drugs commonly used to treat gastric ulcers can cause serious side effects (Yu et al. 2020). The concept of food and medicine sharing the same origin, owing to its simplicity and safety, is regarded as playing a significant role in the prevention and protection against gastric mucosal damage, distinguishing it from conventional drug interventions (Wang et al. 2023).

*Hericium erinaceus* ( *H. erinaceus* ) is a common medicinal and edible fungus, which is rich in a variety of active components. In previous studies, polysaccharides, diterpenes, alkaloids, peptides, and nucleosides have been isolated and identified from *H. erinaceus* (Chen et al. 2019; Cui et al. 2023; Yuan and Liu 2024; Yuan and Liu 2025), which have been shown to play a role in nourishing the spleen and stomach, enhancing immunity and preventing Alzheimer's disease (Tzeng et al. 2018; Li et al. 2024; Shi et al. 2024). In recent years, integrating gut microbiome and metabolomics analysis has become an important strategy for revealing the pharmacological mechanisms of traditional Chinese medicine (Feng et al. 2022; Liu et al. 2022). Dysbiosis of gut microbiota is closely related to the occurrence and development of Gastric ulcer (Li et al. 2022), and metabolic products can play a role in inflammatory responses and immune regulation. Previous studies have reported the protective activity of *H. erinaceus* on the stomach. For example, polysaccharide from *H. erinaceus* has gastric protective activity and antioxidant activity against ethanol‐induced gastric mucosal lesions and pyloric ligation‐induced gastric ulcers (Wang et al. 2018). The polysaccharides of *H. erinaceus* purified from *H. erinaceus* have a pre‐protective effect on ethanol‐induced gastric mucosal injury in rats (Chen et al. 2020). Although the direct antioxidant and mucoprotective effects of *H. erinaceus* are well established, whether these benefits depend on gut microbiota remodeling and downstream metabolic reprogramming remains uncharacterized. Clarifying this host–microbiota–metabolite interaction is critical to advance *H. erinaceus* from traditional dietary practice to a scientifically validated functional food for ulcer prevention.

In this study, chemical analysis and multi‐omics techniques were integrated to explore the gastric protective mechanism of *H. erinaceus* extract (HEE). Firstly, the chemical components of its alcohol extract and water extract were identified by UHPLC‐Q‐TOF MS. Subsequently, in the ethanol‐induced rat gastric injury model, its protective efficacy was confirmed by evaluating the gastric injury index and histopathological changes. Furthermore, proteomics, 16S rRNA sequencing and non‐targeted metabolomics were used to systematically analyze the dynamic changes of gastric tissue, intestinal flora, and serum metabolites. Through multi‐omics joint analysis, the core position of the “gut microbiota–metabolites” axis in mediating gastric protection was revealed.

## 2 Materials and Methods

### 2.1 Materials

*H. erinaceus* (Zhejiang Tianquan Biotechnology Co. LTD, Specimen number: 20231009NY‐07), Ethanol (Aladdin, E111964), Formic acid (Sigma—Aldrich, Merck KGaA, 533,002), Acetonitrile (Sigma—Aldrich, Merck KGaA, 100,029), Ranitidine (Aladdin, R304317), BCA protein assay kit (Beyotime, P0010S), CXCL7 Mouse monoclonal antibody (CLOUD‐CLONE CORP. WUHAN, UMAA370Ra22), GAPDH Polyclonal antibody (Proteintech, 10,494–1‐AP), HRP‐conjugated Goat Anti‐Rabbit IgG (H + L) (Proteintech, SA00001‐2), and Mouse Anti‐Rabbit IgG (CST, #93702).

### 2.2 Preparation of HEE

*H. erinaceus* (Specimen No. 20231009NY‐07) was authenticated by Prof. Hongfei Lv (Zhejiang Sci‐Tech University, Hangzhou, China). The extraction was conducted as previously described, with slight modifications (Lakshmanan et al. 2016; Lv et al. 2021). Fresh *H. erinaceus* fruiting bodies were sectioned and dried in a hot‐air oven (DHG‐9140; Shanghai Yiheng, Instrument Co. Ltd., Shanghai, China) at 50°C for 16 h, then milled through a 120‐mesh sieve. The powder was further dried at 50°C to constant weight and stored desiccated until use. To prepare the water extract (WEHE), powder was suspended in purified water (1:15, w/v), sonicated (50°C, 600 W, 20 min), and hot‐water extracted at 80°C for 1.5 h. The slurry was filtered, and the filtrate was concentrated under reduced pressure, followed by lyophilization. For the alcohol extract (AEHE), powder was mixed with 60% (v/v) ethanol (1:15, w/v) and sonicated under identical conditions for 30 min. The mixture was centrifuged (4000 rpm, 10 min), and the supernatant was retained. The residue underwent a second extraction under identical conditions; supernatants were pooled, concentrated under reduced pressure at 45°C, and lyophilized. Both extracts were stored at −20°C in the dark prior to use.

### 2.3 UHPLC‐Q‐TOF MS Identification of Components in HEE

UHPLC‐Q‐TOF MS analysis was performed following the protocol established (Yang, Wang, et al. 2021; Yang, Mao, et al. 2021), with modifications to optimize the separation conditions. The components of HEE were analyzed by using ACQUITY UPLCTM I‐Class and XevoG3XS LC, and the column was Waters BEH T3 1.8 m (2.1 mm × 150 mm). Mobile phase consisting of 0.1% formic acid (A) and acetonitrile (B), gradient elution conditions: 0–5 min, 5% B; 5–15 min, 5%–30% B; 15–20 min, 30%–45% B; 20–30 min, 45%–80% B; 30–45 min, 80%–95% B; 45.1–55 min, 95%–5% B. Flow rate was 0.3 mL/min. The injection volume was 10 μL, and the column temperature was 40°C. Positive ion mode test was used, and the collection mass range was 50–1200 Da. The capillary voltage was 3 kV; ion source temperature 100°C; atomizer temperature 280°C; atomizer flow rate 800 L/h; cone hole voltage 40 V; and collision energy low energy 6 V/high energy 20–30 V. The data processing software is UNIFI 1.9.2, and the database is Waters Traditional Medicine.

### 2.4 Ethanol‐Induced Acute Gastric Ulcer in Rats

Adult SPF grade SD rats (200 ± 20 g) were obtained from Shanghai BK Laboratory Animal Co. Ltd. All experimental procedures received ethical approval from the Institutional Animal Care and Use Committee (IACUC) of Zhejiang Chinese Medical University Laboratory Animal Research Center (Approval No. IACUC‐20231204‐03) and were conducted in strict accordance with the ARRIVE 2.0 guidelines and national regulations for laboratory animal welfare.

A cohort size of 6 rats per group was adopted, consistent with established protocols for the ethanol‐induced acute gastric injury model (Yun et al. 2017; Zhang et al. 2022). Rats were randomized into 7 groups using a computer‐generated random number sequence. The groups comprised: normal control, model control, positive control (ranitidine, 30 mg/kg) (Zhou et al. 2021), low‐dose AEHE (AE‐L, 150 mg/kg), high‐dose AEHE (AE‐H, 250 mg/kg), low‐dose WEHE (WE‐L, 150 mg/kg), and high‐dose WEHE (WE‐H, 250 mg/kg). These doses were selected on the basis of prior safety and efficacy profiles (Wong et al. 2013; Lakshmanan et al. 2016), and correspond to human equivalent doses within safe dietary supplementation ranges, aligning with traditional consumption patterns. Following a 7‐day acclimatization period, rats received daily oral gavage for 21 days. The control and model groups received purified water, whereas treatment groups received their respective extracts. Investigators performing macroscopic ulcer scoring and histopathological evaluation were blinded to group assignment throughout data collection. After the final administration, rats were fasted for 24 h with free access to water. Acute gastric injury was induced by oral administration of absolute ethanol (5 mL/kg) to all groups except the normal control. One hour post‐ethanol, rats were euthanized under anesthesia. Blood, intestinal contents, and major organs (heart, liver, spleen, lung, kidney, stomach) were collected for subsequent analysis.

#### 2.4.1 Safety Evaluation of HEE

To evaluate the in vivo safety of HEE, this study systematically monitored changes in rat body weight and analyzed major organ indices. Body weights were recorded weekly for 3 weeks. At the conclusion of the experimental period, the animals were euthanized, and the heart, liver, spleen, lung, and kidney were promptly excised. The wet weights of these organs were determined using a precision electronic balance, and the calculation formula for organ indicators is as follows: organ wet weight/body weight*100 (Li et al. 2014).

#### 2.4.2 Macroscopic Assessment of Gastric Injury

After ligating the pylorus, the stomach was cut along the greater curvature and washed with cold saline. The stomach was laid flat to observe and measure the bleeding points and bleeding bands on the surface of the gastric mucosa, record their lengths and widths respectively, and score the injuries according to the established standards. The scoring criteria are as follows: 1 point for bleeding point; 1 point for hemorrhage band length 0–1 mm, 2 points for 1–2 mm, 3 points for 2–3 mm, 4 points for 3–4 mm, 5 points for greater than 5 mm; 1 point for hemorrhage band width 1–2 mm, 2 points for greater than 2 mm. The gastric tissue lesion indices of rats = bleeding point score + length score + width score. The inhibition rates of gastric tissue lesions in rats = (gastric tissue lesion indices in the model group—gastric tissue lesion indices in the experimental group)/gastric tissue lesion indices in the model group (Wang et al. 2025).

#### 2.4.3 Hematoxylin and Eosin (H&E) Staining Analysis of Gastric Tissue Lesions in Rats

H&E staining was used to analyze gastric tissue lesions in rats. The most severe ulcer sites of gastric tissue were cut off and fixed in 10% formaldehyde solution. Subsequently, the tissue was buried in paraffin and prepared into 4 μm tissue sections. The tissue sections were stained with H&E, and then observed under a light microscope to evaluate the pathological changes of gastric mucosa (Wong et al. 2013).

### 2.5 Proteomics Analysis of Gastric Tissue in Rats

Proteins were extracted from gastric tissue of rats by lysate and quantified by BCA. 20 μg of protein was added to loading buffer, incubated in boiling water for 5 min, and separated by SDS‐PAGE electrophoresis (4%–20% precast gradient gel, constant voltage 180 V, 45 min). Proteins were visualized by Coomassie Brilliant Blue R‐250 staining. An appropriate amount of protein from each sample was digested with trypsin using the Filter‐Aided Proteome Preparation method. Peptides were desalted using C18 Cartridges and lyophilized. The lyophilized peptides were reconstituted in 40 μL of 0.1% formic acid solution, and peptide concentration was quantified by measuring the optical density at 280 nm. Peptides from each sample were separated using the NanoElute nanoflow HPLC system. Mobile phase A consisted of 0.1% formic acid in water, and mobile phase B consisted of 0.1% formic acid in acetonitrile. The analytical column (Thermo Scientific EASY column, 25 cm length, 75 μm inner diameter, 1.9 μm particle size) was equilibrated with 95% mobile phase A prior to sample loading. Peptides were separated on the C18 reversed‐phase column at a flow rate of 300 nL/min. Chromatographically separated peptides were analyzed online using a timeTOF Pro mass spectrometer operated in positive ion mode. The ion source voltage was set to 1.5 kV (Wiśniewski et al. 2009; Meier et al. 2018). Both MS and MS/MS spectra were acquired using the time‐of‐flight analyzer, with a mass scan range of 100–1700 m/z. Protein identification and quantification were performed using MaxQuant (Cox and Mann 2008). Peptide and protein false discovery rates (FDR) were controlled at ≤ 0.01. KEGG pathway annotations were performed on the target protein collection using the KOBAS software (Kanehisa et al. 2012).

### 2.6 Western Blotting

Western blot assay was conducted as previously described (Syed et al. 2024). The gastric tissue was quick‐frozen in liquid nitrogen, and then RIPA buffer containing protease inhibitors was added and ground until no obvious tissue blocks were found. Then, it was placed on ice for 30 min of lysis, centrifuged at 4°C and 12,000 rpm for 10 min, and the supernatant was collected. The protein concentration was determined by the BCA method. Equal amounts of proteins were separated by SDS‐PAGE and then electrotransferred to PVDF membranes. After blocking, the membrane was incubated overnight with the primary antibodies CXCL7 (1:1000) and GAPDH (1:5000) at 4°C, and then incubated at room temperature with the corresponding HRP‐labeled secondary antibody for 2 h. After exposure to ECL chemiluminescence reagent, the protein bands were quantitatively analyzed using ImageJ software.

### 2.7 Metabolomics Analysis of Serum in Rats

The samples were thawed at 4°C and 100 μL aliquots were mixed with 400 μL of cold methanol/acetonitrile (1:1, v/v) to remove the protein. The mixture was centrifuged for 20 min (14,000 g, 4°C). The supernatant was dried in a vacuum centrifuge. For LC–MS analysis, the samples were re‐dissolved in 100 μL acetonitrile/water (1:1, v/v) solvent and centrifuged at 14000 g at 4°C for 15 min, then the supernatant was injected. Analysis was performed using a UHPLC (Vanquish UHPLC, Thermo) coupled to an Orbitrap Exploris 480 in Shanghai Applied Protein Technology Co. Ltd. The ESI source conditions were set as follows: Ion Source Gas1 as 50, Ion Source Gas2 as 2, source temperature: 350°C, IonSpray Voltage Floating (ISVF): +3500 V/‐2800 V. In MS only acquisition, the instrument was set to acquire over the m/z range 70–1200 Da, the resolution was set at 60000, and the accumulation time was set at 100 ms. In auto MS/MS acquisition, the instrument was set to acquire over the m/z range 70–1200 Da, the resolution was set at 60000, the accumulation time was set at 100 ms, and the exclusion time was 4 s. The raw MS data were converted to MzXML files using ProteoWizard MSConvert before importing into freely available XCMS software. For peak picking, the following parameters were used: centWave m/z = 10 ppm, peakwidth = c (10, 60), prefilter = c (10, 100). For peak grouping, bw = 5, mzwid = 0.025, minfrac = 0.5 were used. CAMERA (Collection of Algorithms of MEtabolite pRofile Annotation) was sued for annotation of isotopes and adducts. In the extracted ion features, only the variables having more than 50% of the nonzero measurement values in at least one group were kept (Huang et al. 2013; Blaženović et al. 2018; Contrepois et al. 2020). Compound identification of metabolites was performed by comparing of accuracy m/z value (< 10 ppm), and MS/MS spectra with an in‐house database established with available authentic standards.

### 2.8 16S rRNA Amplicon Sequencing Analysis of Intestinal Contents

Total genome DNA from samples was extracted using the Mag‐bind soil DNA kit (Omega), and tests the purity and concentration of DNA. According to the selection of the sequencing region, the selected V3‐V4 variable region was amplified by PCR using specific primers with a barcode and high‐fidelity DNA polymerase. PCR products were detected by 2% agarose gel electrophoresis, and the target fragments were cut and recovered by the Quant‐iT PicoGreen dsDNA Assay Kit. Referring to the preliminary quantitative results of electrophoresis, the PCR amplification recovered products were detected and quantified with the Microplate reader (BioTek, FLx800) fluorescence quantitative system, and the corresponding proportions were mixed according to the sequencing requirements of each sample. The library was constructed using the TruSeq Nano DNA LT Library Prep Kit from Illumina. The constructed library is inspected by the Agilent Bioanalyzer 2100 and Promega QuantiFluor. After the library is qualified, it is sequenced (Lozupone and Knight 2005; Caporaso et al. 2011; Avershina et al. 2013).

### 2.9 Statistical Analysis

All data are expressed as mean ± standard deviation (SD) of at least three independent experiments. The student *t*‐test was used for comparing two conditions, and one‐way analysis of variance (ANOVA) with Tukey's test was used for multiple comparisons. All analyses were performed using GraphPad Prism 8.0, with a *p* < 0.05 considered statistically significant.

## 3 Results

### 3.1 Analysis of the Composition of HEE

The WEHE was analyzed by ultra‐high performance liquid chromatography and triple quadrupole time‐of‐flight mass spectrometry. A total of 88 components were detected, including 25 terpenes, 21 alkaloids, 11 phenols, 4 ketones, and 3 nucleosides. In the AEHE, a total of 215 components were detected, including 64 terpenes, 21 alkaloids, 20 fatty acids, 13 phenols, and 8 flavonoids. Detailed information on these compounds was provided in Tables S1 and S2. There are 49 common components, including adenosine, cnidilide, cycloleucine, delphamine, and estriol in the WEHE and AEHE.

### 3.2 HEE Attenuates Ethanol‐Induced Acute Gastric Injury

**FIGURE 1:**
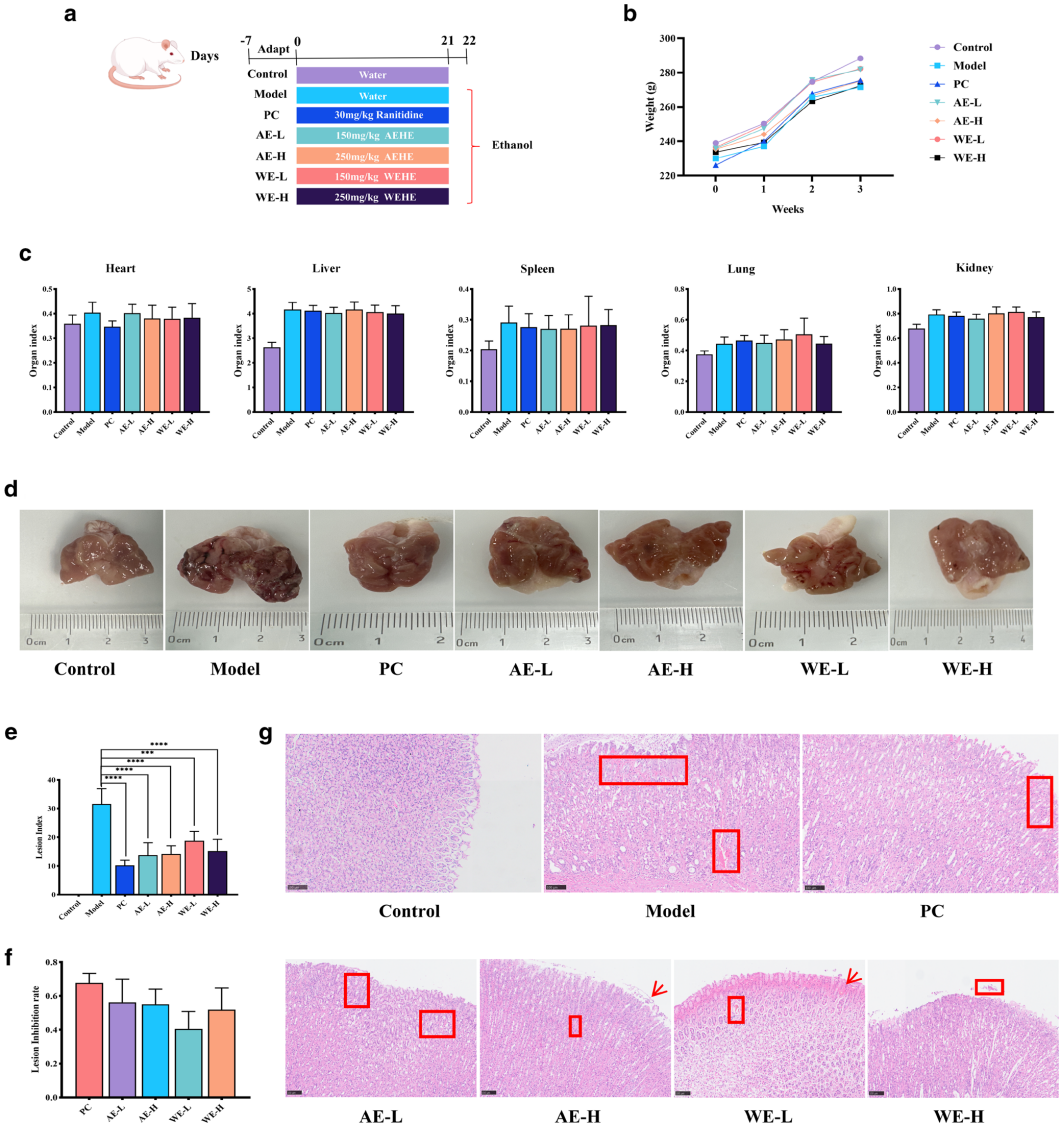
HEE prevents ethanol‐induced acute gastric injury caused by ethanol. (a) Schematic diagram of the experimental design (*n* = 6). (b) Body weight changes. (c) Organ indices. (d) Gastric tissue image. (e) Lesion index of the gastric. (f) Lesion inhibition rate of the gastric. (g) Representative HE staining of gastric, scale bar: 100 μm.

To evaluate safety, body weights were recorded weekly for 3 weeks (Figure 1b). No significant differences were observed between the model and treatment groups. Similarly, organ indices (heart, liver, spleen, lung, and kidney) did not differ significantly among groups (Figure 1c). These findings indicate that AEHE and WEHE exhibit no observable toxicity and maintain normal physiological status in rats.

To evaluate the intervention effect of HEE on ethanol‐induced acute gastric injury in rats, this study conducted an analysis through gastric tissue morphology observation, damage index calculation, and pathological section staining. After dissection, the gastric organs of rats in each group were incised (Figure 1d), and the gastric tissue damage index was calculated on the basis of the length and width of bleeding spots and bleeding bands (Figure 1e). Compared to controls, model rats exhibited severe gastric bleeding and elevated damage indices, confirming successful model establishment. All interventions significantly reduced gastric damage indices (*p* < 0.001, *p* < 0.0001), with inhibition rates of 56.32%, 55.06%, 40.50%, 51.89%, and 67.72% for low‐ and high‐dose AEHE, low‐ and high‐dose WEHE, and the positive control (PC), respectively (Figure 1f). H&E staining was used to observe the pathological changes of rat gastric tissue, and the results are shown in Figure 1g: no obvious lesions were found in the gastric tissue of the blank control group; the model group showed severe gastric mucosal bleeding and inflammatory cell infiltration, which was significantly different from the blank control group, further verifying the successful construction of the rat acute gastric ulcer model. In the AE‐L group, AE‐H group, WE‐L group, WE‐H group, and PC group, inflammatory cell infiltration was observed, but the number was significantly lower than that in the model group. In conclusion, AEHE and WEHE have significant preventive and protective effects on ethanol‐induced acute gastric injury in rats.

### 3.3 Effect of HEE on Gastric Protein Expression in Rats With Ethanol‐Induced Acute Gastric Injury

**FIGURE 2:**
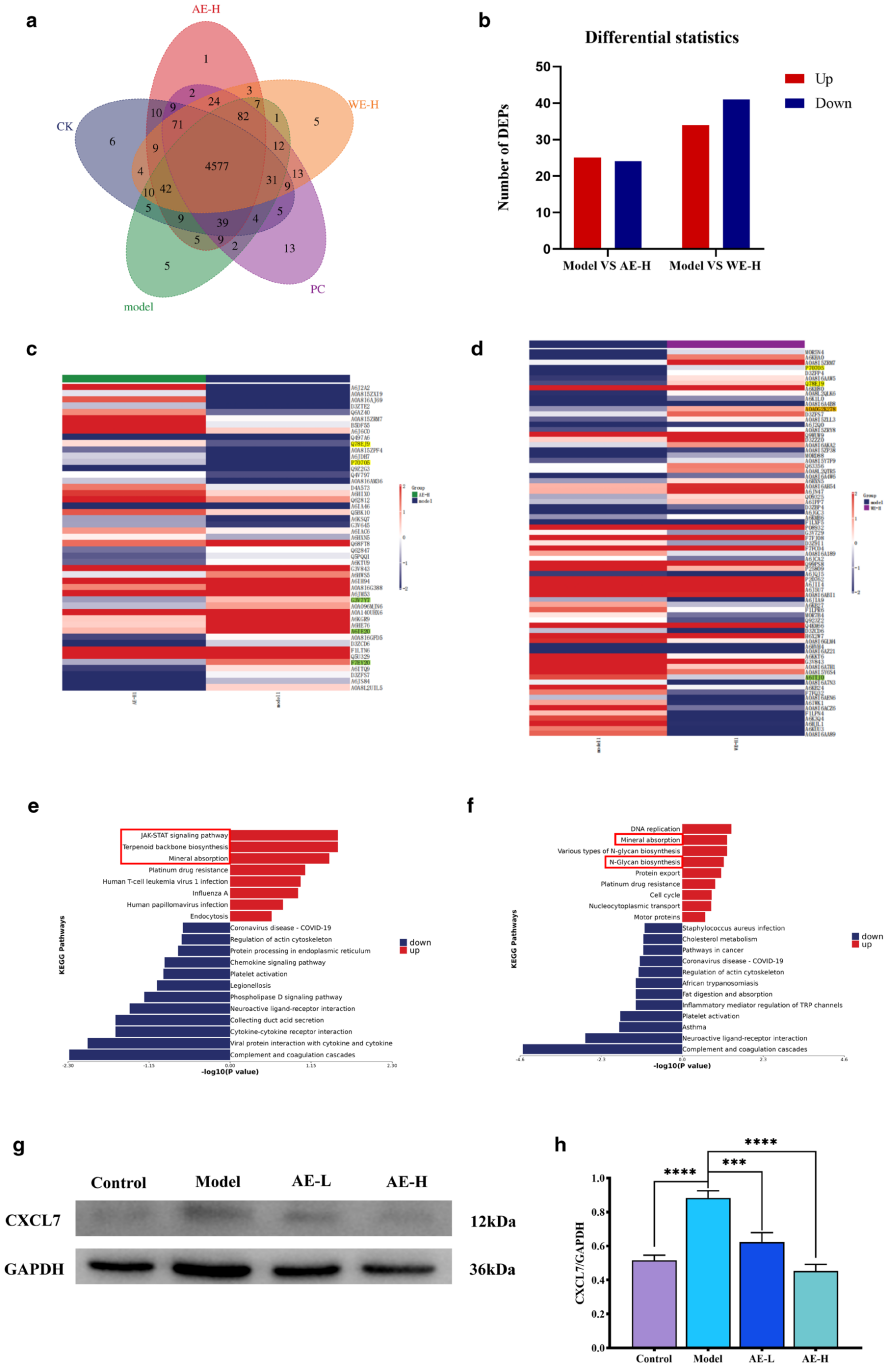
Effects of HEE on gastric protein expression. (a) Venn diagram of proteins across the control, Model, PC, AE‐H, and WE‐H groups. (b) Bar chart of DEPs between Model vs. AE‐H and Model vs. WE‐H groups. (c) Heatmap of DEPs in AE‐H group (up‐ and down‐regulated). (d) Heatmap of DEPs in WE‐H group (up‐ and down‐regulated). (e) Pathway enrichment butterfly plot of DEPs (Model vs. AE‐H). (f) Pathway enrichment butterfly plot of DEPs (Model vs. WE‐H). (g) Immunoblot bands of CXCL7. (h) Quantitative analysis of CXCL7/GAPDH ratio. (Compared with the Model group, *p < 0.05, **p < 0.01, ***p < 0.001).

Proteomic analysis was conducted on the gastric samples of the control group, model group, AE‐H group, and WE‐H group. By comparing the identification results of different groups through the Venn diagram (Figure 2a), the overlap and differences among them have been visually presented. Differentially expressed proteins (DEPs) were defined using thresholds of fold change > 2 and *p* < 0.05. Relative to the model group, AE‐H treatment yielded 25 upregulated and 24 downregulated DEPs, whereas WE‐H treatment identified 34 upregulated and 41 downregulated DEPs (Figure 2b). In the AE‐H group, chemokines CXCL7 (F7EY20), caveolin (A6IE20), and sodium/hydrogen exchange (G3V7Y7) were significantly downregulated. In the WE‐H group, glutathione peroxidase (GPx) (A0A0G2K278) was significantly upregulated, and phospholipase A2 (A6ITJ0) was significantly downregulated. The protein expression levels of Calpain‐8 protein (Q78EJ9) and Copper‐transporting ATPase 1 (P70705) were upregulated in both the AE‐H and WE‐H groups (Figure 2c,d, Tables S3 and S4). All significantly different proteins were annotated by the KEGG pathway. The significantly upregulated differential proteins in the AE‐H group were mainly enriched in the JAK–STAT signaling pathway, terpene skeleton biosynthesis, and mineral absorption pathway (Figure 2e). Compared with the model group, the significantly upregulated differential proteins in the WE‐H group were mainly enriched in the mineral absorption and N‐glycan biosynthesis pathways (Figure 2f).

### 3.4 AEHE Reduces the Expression of Chemokine CXCL7 and Alleviates Gastric Inflammation

Proteomic analysis suggests that AEHE may exert gastric protective effects by down‐regulating the expression of the chemokine CXCL7. CXCL7 is known to recruit inflammatory cells to the site of injury, thereby exacerbating tissue inflammation (Wu et al. 2022). We validated protein levels via Western blotting. Model rats exhibited significantly elevated CXCL7 expression compared to controls. However, treatment with AEHE significantly suppressed CXCL7 levels in a dose‐dependent manner (Figure 2g,h). These findings confirm that AEHE inhibits ethanol‐induced CXCL7 upregulation, thereby mitigating inflammatory infiltration and tissue damage.

### 3.5 HEE Regulates the Content of Serum Metabolites in Rats With Ethanol‐Induced Acute Gastric Injury

**FIGURE 3:**
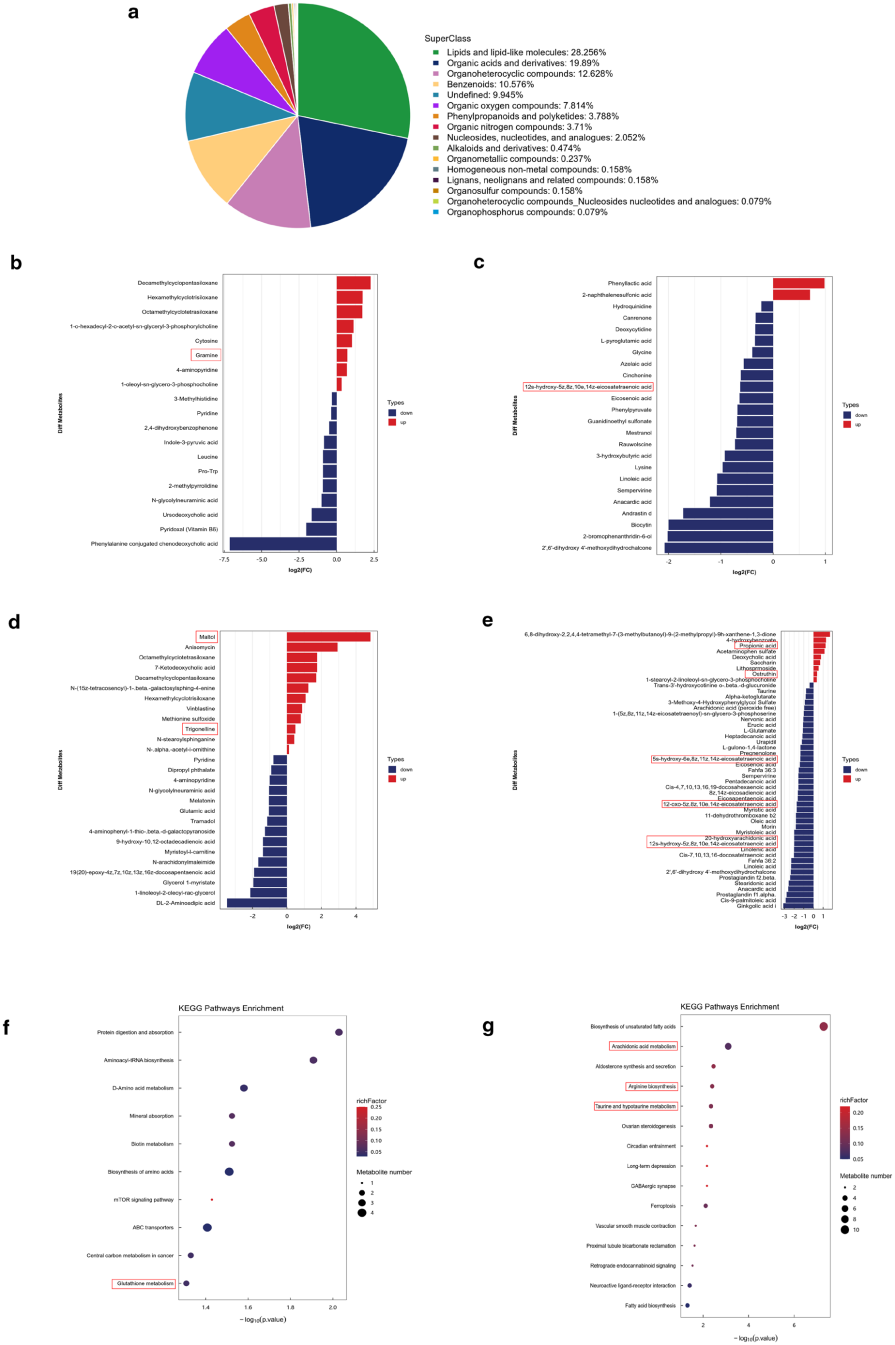
Effects of HEE on serum metabolome results. (a) Pie chart of metabolite chemical classifications. (b) Butterfly plot of differential metabolites between Model and AE‐H groups (positive ion mode). (c) Butterfly plot of differential metabolites between Model and AE‐H groups (negative ion mode). (d) Butterfly plot of differential metabolites between Model and WE‐H groups (positive ion mode). (e) Butterfly plot of differential metabolites between Model and WE‐H groups (negative ion mode). (f) Bubble plot of the top 10 enriched KEGG pathways (AE‐H group). (g) Bubble plot of the top 10 enriched KEGG pathways (WE‐H group).

Serum metabolites identified in both positive and negative ion modes were categorized by chemical classification (Figure 3a). Lipids and lipid‐like molecules, organic acids and derivatives, organo heterocyclic compounds, benzenoids, and organic oxygen compounds are the main components in metabolites. Among them, lipids and lipid molecules account for the highest proportion, reaching 28.26%. On the basis of FC > 1.5 or FC < 0.67 and *p* < 0.05, differential metabolites were screened out.

The visualization results of the significantly different metabolites between the AE‐H group and the model group are shown in Figure 3b,c. In the diagram, red markers represent upregulated differential metabolites, and those in blue represent downregulated ones. In the positive ion detection mode, the differential metabolite gramine was significantly upregulated in the AE‐H group. In the negative ion mode, 12 s ‐hydroxy—5z,8z,10e,14z ‐eicosapentaenoic acid (12 (S)‐HETE) was significantly downregulated. The significant differential metabolites in the WE‐H group and the model group were visualized, as shown in Figure 3d,e. Under the positive ion detection mode, the analysis of differential metabolites between the WE‐H group and the model group revealed that the expression levels of maltol and trigonelline were significantly upregulated. In the negative ion mode, the significantly upregulated metabolites were propionic acid and ostruthin; in addition, several arachidonic acid metabolic derivatives, including 12 (S)‐HETE, 12‐oxygen‐5Z,8Z,10E,14Z‐eicosapentaenoic acid (12‐oxo‐ETE), were significantly downregulated.

KEGG pathway enrichment analysis of differential metabolites revealed that in the AE‐H group, the pathways related to the gastric mucosal protection mechanism were significantly enriched with the glutathione metabolism pathway as the core (Figure 3f). The gastric protective pathways in the WE‐H group presented the characteristics of multi‐pathway synergistic enrichment, specifically including the arachidonic acid metabolic pathway, the arginine biosynthesis pathway, and the taurine and hypotaurine metabolic pathways (Figure 3g).

### 3.6 HEE Regulates the Gut Microbiota in Rats With Ethanol‐Induced Acute Gastric Injury

**FIGURE 4:**
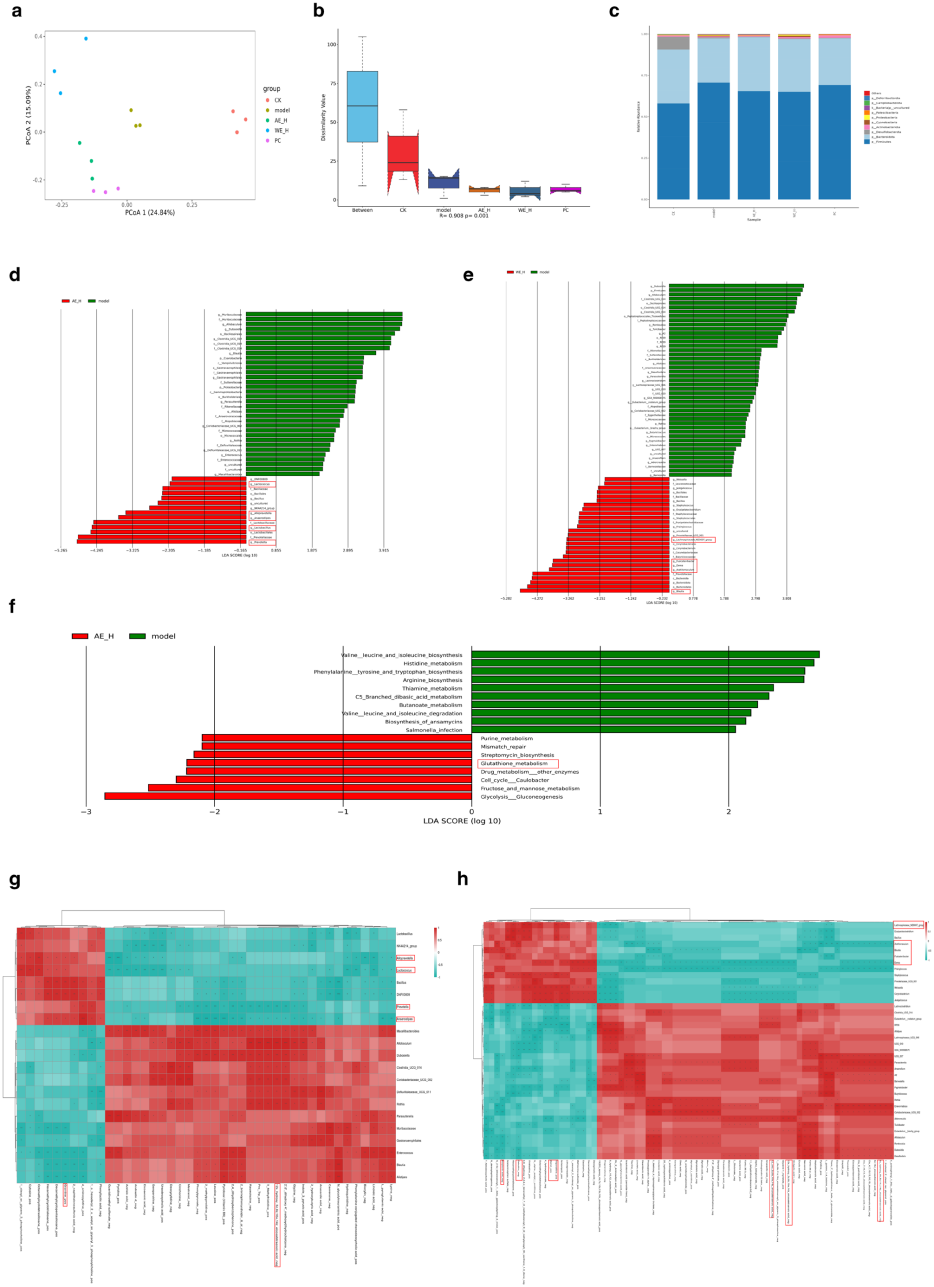
Alterations in the composition and function of gut microbiota induced by HEE. (a) PCoA plot on the basis of Bray–Curtis Distance. (b) Boxplot for intergroup differences on the basis of Bray–Curtis Distance Anosim analysis. (c) Relative species abundance at phylum level. (d) LDA identified differentially enriched genera (Model vs. AE‐H). (e) LDA identified differentially enriched genera (Model vs. WE‐H). (f) KEGG functional enrichment differences (Model vs. AE‐H). (g) Spearman correlation heatmap of differential flora and metabolites (Model vs. AE‐H). (h) Spearman correlation heatmap of differential flora and metabolites (Model vs. WE‐H).

The Bray–Curtis‐based principal coordinate analysis (PCoA) showed significant clustering of gut microbiota in each group (Figure 4a). ANOSIM analysis further validates the presence of highly significant intergroup differences in the composition of the gut microbiota (*R* = 0.908, *p* < 0.001) (Figure 4b). The Firmicutes/Bacteroidetes ratio (F/B ratio) in the model group was significantly higher than that in the control group. Compared with the model group, AEHE and WEHE supplementation resulted in a reversal of the F/B ratio (Figure 4c). At the genus level, compared with the model group, the relative abundance of genera such as *Prevotella*, *Lactobacillus*, *Anaerostipes*, *Alloprevotella*, and *Lactococcus* in the AE‐H group was upregulated (Figure 4d). In the WE‐H group, genera such as *Blautia*, *Dorea*, *Acetitomaculum*, *Fusicatenibacter*, and *Lachnospiraceae*_ND3007_group also showed an upward trend (Figure 4e). All these different genera of bacteria belong to short‐chain fatty acids (SCFA)‐producing bacteria (Shi et al. 2021; Lin et al. 2022; Nguyen et al. 2023; Park et al. 2023; Mao et al. 2024; Kim et al. 2025; Kovynev et al. 2025; Meiners et al. 2025; Yoo et al. 2025; Zou et al. 2025). To further understand the functional potential of these microbial communities, PICRUSt was applied to predict the composition of functional genes. KEGG functional prediction analysis showed that the glutathione metabolic pathway was significantly upregulated in the AE‐H group compared with the model group (Figure 4f).

### 3.7 Integrated Analysis of 16S rRNA Amplicon Sequencing and Non‐Targeted Metabolomics

An integrated analysis of 16S rRNA sequencing and metabolomics revealed that in the AE‐H group, the up‐regulation of the metabolite gramine exhibited a significant positive correlation with *Anaerostipes, Alloprevotella*, and *Lactococcus* (*p* < 0.05). Conversely, the downregulation of the metabolites 12 (S)‐HETE was significantly and negatively correlated with *Prevotella* and *Anaerostipes* (*p* < 0.05) (Figure 4g). In the WE‐H group, the gut microbiota *Dorea* exhibited a significant positive correlation with the metabolite maltol (*p* < 0.05). Additionally, the *Lachnospiraceae*_ND3007_group showed a significant positive association with trigonelline (*p* < 0.01). We observed that there was a highly significant positive correlation between propionic acid concentration and the abundance of the genus *Blautia* (*p* < 0.01), and also a significant positive correlation with the genera *Acetitomaculum* and *Fusicatenibacter* (*p* < 0.05). Ostruthin was extremely significantly positively correlated with the genus *Acetitomaculum* (*p* < 0.01), and significantly positively correlated with the genera *Lachnospiraceae_*ND3007_group and *Blautia* (*p* < 0.05). Further analysis indicated that various arachidonic acid metabolic derivatives displayed a similar significant negative correlation trend with *Dorea* (Figure 4e). These results indicate that AEHE and WEHE treatments can significantly enhance the protective effect on the gastric mucosa through the bacteria‐metabolite interaction network.

## 4 Discussion

Our findings demonstrate that HEE significantly attenuates ethanol‐induced gastric injury in rats, an effect mediated by coordinated modulation of gut microbiota composition and host metabolic profiles. Gastric ulcers clinically present with postprandial epigastric pain, nausea, and anorexia (Gong et al. 2024), and the gastroprotective properties of *H. erinaceus* have long been documented in traditional Chinese medicine. Prior studies confirmed that aqueous and ethanolic fractions mitigate acute ethanol‐induced gastric injury (Wong et al. 2013; Lv et al. 2021). However, the precise mechanisms underlying the interplay between gut microbiota remodeling and host metabolic reprogramming remain incompletely understood. By integrating 16S rRNA sequencing with metabolomic profiling, we identified specific gut microbiota and their derived metabolites associated with HEE treatment. These data map a multi‐pathway mechanism underlying HEE‐mediated gastric protection and gut microbiome remodeling, substantiating its application in functional food designed for gastric health maintenance.

Both AEHE and WEHE significantly attenuated ethanol‐induced acute gastric injury in rats, as evidenced by reduced ulcer indices, diminished hemorrhage, and suppressed inflammatory cell infiltration, with no observable systemic toxicity. Proteomic analysis of gastric tissues revealed upregulated Calpain‐8 and copper‐transporting ATPase in both treatment groups compared to the model. The enhanced Calpain‐8 expression suggests improved membrane trafficking in surface mucous cells, bolstering mucosal defense against stress. Concurrently, elevated copper‐transporting ATPase implies modulated copper metabolism, potentially supporting antioxidant enzyme function (Lutsenko et al. 2007). Beyond these common pathways, AEHE dose‐dependently downregulated CXCL7, as confirmed by Western blotting. Since model rats displayed elevated CXCL7 levels relative to controls, AEHE‐mediated suppression likely attenuates chemokine‐driven leukocyte infiltration, thereby mitigating focal mucosal injury. Simultaneously, WEHE exhibited significant upregulation of GPx, complemented by KEGG enrichment of the mineral absorption pathway. Given selenium's role as a GPx cofactor (Hariharan and Dharmaraj 2020), this pathway activation likely supports GPx‐mediated ROS scavenging and redox homeostasis (Shen et al. 2022). In short, the distinct anti‐inflammatory and antioxidant effects of these extracts combine to protect and repair gastric tissue, supporting their development as functional food ingredients for gastrointestinal care.

Non‐targeted metabolomics profiling revealed distinct metabolic alterations in rats with acute ethanol‐induced gastric injury following HEE treatments. AEHE treatment significantly increased gramine levels while suppressing 12 (S)‐HETE accumulation. Gramine concurrently attenuates NF‐κB signaling by blocking p105 ubiquitination and enhances SOD and catalase activities (Dao et al. 2024), whereas reduction of 12 (S)‐HETE, a pro‐inflammatory eicosanoid that drives leukocyte infiltration (Kulkarni et al. 2021), likely limits localized tissue damage. KEGG pathway analysis further indicated significant enrichment of glutathione metabolism in the AE‐H group. This metabolic activation aligns with functional profiles predicted from 16S rRNA gene sequencing data, reinforcing the consistency between microbial potential and metabolic outcomes. In contrast, WEHE administration elevated maltol, trigonelline, propionic acid, and ostruthin. These compounds collectively reinforce mucosal defense: maltol engages the PI3K/Akt‐Nrf2/HO‐1 axis (Lee et al. 2025), trigonelline boosts glutathione‐dependent antioxidant capacity while limiting lipid peroxidation (Khalili et al. 2018), propionic acid modulates immune homeostasis as a microbiota‐derived short‐chain fatty acid (Sun et al. 2017), and ostruthin suppresses iNOS and COX‐2 expression (Tuan Anh et al. 2017). The synergistic up‐regulation of the above metabolites suggests that HEE treatment may exert gastric protective effects through a multi‐target and multi‐pathway mechanism.

Ethanol exposure broadly disrupted microbiota structure, increasing the F/B ratio associated with inflammatory diseases (Spychala et al. 2018; Wang et al. 2022; Petakh et al. 2023). HEE intervention normalized this ratio, suggesting restored microbial homeostasis. More critically, HEE enriched SCFA‐producing taxa, whose metabolic output likely promotes Nrf2 nuclear translocation via histone deacetylase (HDAC) inhibition, thereby upregulating endogenous antioxidant defenses (González‐Bosch et al. 2021). These microbial metabolites concurrently suppress NF‐κB signaling, downregulating pro‐inflammatory mediators such as TNF‐α and IL‐6 (Segain et al. 2000). KEGG analysis further confirmed activated glutathione metabolism in the AEHE group, critical for mitigating oxidative stress via ROS scavenging and protein repair (Yang, Wang, et al. 2021; Yang, Mao, et al. 2021). These findings establish a foundational framework for understanding how HEE protects gastric mucosa. To elucidate the specific microbial drivers behind these changes, we further analyzed correlations between key microbiota and metabolites.

Multi‐omics integration revealed extract‐specific microbiota‐metabolite networks underlying HEE's gastroprotective effects. In the AEHE group, gramine levels positively correlated with *Anaerostipes*, *Alloprevotella*, and *Lactococcus*. As a natural indole alkaloid, gramine exhibits documented antioxidant and anti‐inflammatory properties (Ramu et al. 2018; Sabi et al. 2024). Functionally, *Anaerostipes* is a butyrate‐producing genus (Bui et al. 2014), but butyrate is critical for maintaining gastric mucosal barrier integrity and suppressing inflammation (Venkatraman et al. 2025). Similarly, *Lactococcus*, classified as lactic acid bacteria, includes strains with known immunomodulatory functions (Okuno et al. 2025). On the basis of these correlations and the functional characteristics of the microbiota, we hypothesize that AEHE promotes the colonization of these beneficial genera by modulating gramine production, thereby creating a favorable gut microenvironment for gastric mucosal repair. Conversely, WEHE treatment established a robust propionate‐*Blautia* association (*p* < 0.01). As a core member of the mammalian gut microbiota within the *Firmicutes* phylum (Durand et al. 2017; Liu et al. 2021), *Blautia* is a producer of SCFAs, including acetate and propionate (Durand et al. 2017; Payen et al. 2020), via carbohydrate metabolism. Beyond metabolite production, *Blautia* enrichment is associated with reduced inflammatory diseases and metabolic syndrome (Liu et al. 2021; Thu Thuy Nguyen and Endres 2022). Propionate modulates inflammatory responses by regulating epithelial and leukocyte immune functions, specifically influencing chemotaxis, cytokine synthesis, and free radical generation (Vinolo et al. 2011). Collectively, these data suggest that WEHE exerts therapeutic effects on gastric ulcers by enriching *Blautia*, thereby driving propionic acid production to mitigate inflammation. A positive correlation emerged between *Fusicatenibacter* abundance and propionic acid levels. Although *Fusicatenibacter* is not definitively proven to produce propionate, this pattern corroborates broader evidence that dietary fiber modulates similar microbiota to influence metabolic profiles (Liang et al. 2023). Thus, the observed enrichment may reflect a conserved regulatory mechanism rather than direct synthesis. Ostruthin levels correlated positively with the abundance of SCFA‐producing bacteria, including *Acetitomaculum*, *Blautia*, and *Lachnospiraceae*_ND3007_group. Although the antioxidant and anti‐inflammatory activities of ostruthin are well‐documented (Joa et al. 2011; Tuan Anh et al. 2017), its regulatory effect on gut microbiota remains unexplored. Given that these genera produce SCFAs critical for gut barrier integrity and epithelial repair, we hypothesize that ostruthin promotes their colonization. This enrichment likely enhances SCFA supply, consequently contributing to gastric mucosal repair. Negative correlations were observed between pro‐inflammatory metabolites and specific microbiota across both extracts. Specifically, 12 (S)‐HETE down‐regulation aligned with *Prevotella* and *Anaerostipes* enrichment (AEHE), whereas arachidonic acid derivatives correlated with *Dorea* (WEHE). These findings suggest that enriching these genera may alleviate local inflammatory responses by suppressing pro‐inflammatory mediators like 12 (S)‐HETE. Thus, HEE likely exerts protective effects by modulating this microbiota‐metabolite axis, simultaneously boosting beneficial SCFAs and suppressing harmful mediators like 12 (S)‐HETE.

Although WEHE and AEHE showed similar therapeutic efficacy for gastric ulcer, multi‐omics profiling identified distinct modulatory capacities of WEHE on gut microbiota. Proteomic and metabolomic data suggest that WEHE enhances antioxidant defense via GPx upregulation and mitigates inflammation through elevated propionic acid levels. Notably, propionic acid concentrations correlated positively with *Blautia* and *Fusicatenibacter* abundance in the WEHE group. Collectively, these findings highlight WEHE's potential in modulating gut microbiota and metabolites. Nevertheless, causal validation methods (e.g., antibiotic treatment, fecal microbiota transplantation (FMT), or metabolite supplementation) were not employed in this study; therefore, the direct regulatory effect of microbiota on metabolites cannot be definitively confirmed. The observed correlations may reflect unidirectional, reverse, or bidirectional interactions between microbiota and metabolites. Future investigations using FMT models combined with metabolite intervention experiments are warranted to elucidate the functional mechanisms of specific microbiota and their metabolites in gastric ulcer.

## 5 Conclusion

In the present study, both WEHE and AEHE alleviated acute ethanol‐induced gastric injury, yet multi‐omics profiling identified WEHE as superior in regulating gut microbiota. Mechanistically, WEHE conferred gastric mucosal protection by enriching SCFA‐producing bacteria, specifically *Blautia*, thereby promoting propionic acid production. Collectively, WEHE attenuates gastric injury by modulating the “gut microbiota‐metabolites” axis, involving the enhancement of beneficial metabolites, inhibition of pro‐inflammatory mediators, and regulation of glutathione metabolism. Nevertheless, causal validation methods were not employed in this study. In the future, the FMT model combined with metabolite intervention experiments can be utilized for further research. These findings collectively alleviate oxidative stress and inflammatory damage, offering insights for intervention strategies targeting gut microbiota and metabolic profiles.

## Author Contributions

**Ruixin Bei:** validation, writing – review and editing, writing – original draft, conceptualization, formal analysis. **Tingxuan Zong:** validation, writing – review and editing. **Yanfang Sun:** writing – review and editing, funding acquisition. **Lifan Yu:** methodology, conceptualization, formal analysis, writing – review and editing. **Zhonghua Lu:** methodology, writing – review and editing.

## Funding

This work is supported by Zhejiang Province “San Nong Jiu Fang” Agricultural Science and Technology Collaborative Program “Open Competition Mechanism” Project: Research and Application Demonstration Project of Key Technologies of Preservation and Processing of *Hericium erinaceus* (2023SNJF025‐1).

## Ethics Statement

The authors declare no conflicts of interest. This study was approved by the Institutional Review Board of Zhejiang Chinese Medical University (Approval No. IACUC‐20231204‐03).

## Supporting information


**Table S1:** The composition of WEHE.
**Table S2:** The composition of AEHE.
**Table S3:** Names of DEPs in the WE‐H group.
**Table S4:** Names of DEPs in the AE‐H group.

## Data Availability

The data that support the findings of this study are available from the corresponding author upon reasonable request.
