# Supplementary material for: Hericium erinaceus Extracts Exert Gastroprotective Effects Through Modulation of the Gut Microbiota and Metabolites
Source: Food Sci Nutr. 2026 May 11;14(5):e71851. doi: 10.1002/fsn3.71851 (PMC13158580; doi:10.1002/fsn3.71851)
Supplement: Supplementary file 1 — Table S1: The composition of WEHE. Table S2: The composition of AEHE. Table S3: Names of DEPs in the WE‐H group. Table S4: Names of DEPs in the AE‐H group. [file FSN3-14-e71851-s001.docx]

**Supporting information to *Hericium erinaceus* extracts exert gastroprotective effects through modulation of the gut microbiota and metabolites**

Ruixin Bei^1, 2^, Lifan Yu^1, 2^, Zhonghua Lu^3^, Tingxuan Zong^3^, Yanfang Sun^1, 2, *^

^1^ *College of Life Sciences and Medicine, Zhejiang Sci-Tech University, Hangzhou 310018, China*

^2^ *Zhejiang International Joint Laboratory of Traditional Medicine and Big Health Products Development, Hangzhou 310018, China*

*^3^ Zhejiang Provincial Agricultural Technology Extension Center, Hangzhou 310018, China*

^*^Corresponding authors: Prof. Yanfang Sun

College of Life Sciences and Medicine, Zhejiang Sci-Tech University, Hangzhou 310018, China

Tel.: +86 0571 86843187; Fax: +86 0571 86843187

E-mail address: [katherineyfs@zstu.edu.cn](mailto:katherineyfs@zstu.edu.cn) (Y. Sun)

**Table S1 The composition of WEHE**

| **Name** | **Formula** | **Classification** | **Name** | **Formula** | **Classification** |
| --- | --- | --- | --- | --- | --- |
| (-)-Jasmonic acid | C12H18O3 | Terpenoids | Ethyl 4-hydroxybenzoate | C9H10O3 | Phenols |
| (+)-Curcumenol | C15H22O2 | Terpenoids | Ethyl 5-O-caffeoyl-3-O-sinapoylquinate | C29H32O13 | Organic acid |
| (4e)-1,5-Bis(4-hydroxyphenyl)-2-(hydroxymethyl)-4-penten-1-ol | C18H20O4 | Phenols | Galdosol | C20H24O5 | Terpenoids |
| (4e)-1,5-Bis(4-hydroxyphenyl)-2-(methoxymethyl)-4-penten-1-ol | C19H22O4 | Phenols | Gastrodioside | C20H24O8 | Glycosides |
| (R)-1,5-Dimethylhexylamine | C8H19N | Amide | GelsemAmide | C20H24N2O3 | Alkaloid |
| 1-(3-Methoxy-4- hydroxyphenyl)-4-undecene-3-one | C18H26O3 | Phenols | Gibberellin A119 | C19H24O5 | Terpenoids |
| 1-(4-Hydroxy-3-methoxyphenyl)-7-phenyl-3,5-heptanediol | C20H26O4 | Phenols | Guaia-1(10),11-dien-9-one | C15H22O | Ketones |
| 1,2-Benzothiazole | C7H5NS | Alkaloid | Homosenkyunolide I | C13H18O4 | Phthalides |
| 1,5-Bis(4-hydroxy-3-methoxyphenyl)-1,4-pentadien-3-one | C19H18O5 | Phenols | Humanteniridine | C31H38N2O8 | Alkaloid |
| 11-Methoxyhumantenine | C22H28N2O4 | Alkaloid | Icariside B10 | C19H32O8 | Terpenoids |
| 15-Hydroxyhumantenoxenine | C21H24N2O5 | Alkaloid | Isotetrandrine | C38H42N2O6 | Alkaloid |
| 16alpha-Acetoxyhyoscyamilactol | C30H42O8 | Steroid | Jasmololone | C11H16O2 | Terpenoids |
| 19(E)-Humantenine | C21H26N2O3 | Alkaloid | Kankanoside P | C16H26O8 | Terpenoids |
| 1beta-Glucogeniposide | C17H26O10 | Terpenoids | Kudzusapogenol A | C30H50O5 | Terpenoids |
| 1-Deoxynojirimycin | C6H13NO4 | Alkaloid | Kupitengester 3 | C30H38O11 | Terpenoids |
| 2, 6-dimethoxybenzoquinone | C8H8O4 | Quinones | leeaoside | C24H40O11 | Terpenoids |
| 21-O-Methyl-5-pregnene-3beta,17beta,21-tetrol-20-one | C22H34O5 | Ketones | Mannitol | C6H14O6 | Sugar alcohol |
| 2-Deacetoxy-9-acetoxytaxine B | C33H45NO7 | Alkaloid | Methylbenzylamine | C8H11N | Alkaloid |
| 3,4-Dihydroxycinnamic acid | C9H8O4 | Phenols | Methylparaben | C8H8O3 | Phenols |
| 3-Butyl-4-methylphthalic acid | C13H16O4 | Carboxylic acid | Mussaenosidic acid | C16H24O10 | Terpenoids |
| 5-Methoxy dehydrodiisoeugenol | C21H24O5 | Phenols | Nigakilactone H | C22H32O8 | Terpenoids |
| Adenine | C5H5N5 | Nucleosides | OleAmide | C18H35NO | Fatty amide |
| Adenosine | C10H13N5O4 | Nucleosides | Pariposide D | C39H60O15 | Steroid |
| Ajugacumbin C | C31H42O11 | Terpenoids | Phanginin G | C21H28O5 | Terpenoids |
| Apocynoside II | C19H30O9 | Glycosides | Phanginin J | C21H26O5 | Terpenoids |
| Arctigenin | C21H24O6 | Lignan | Pipercide | C22H29NO3 | Alkaloid |
| Artanomaloide | C32H36O8 | Terpenoids | Prostephabyssine | C19H23NO5 | Alkaloid |
| Aryl-8-oxa-bicyclo[3,2,1]-oct-3-en-2-one | C16H18O6 | Ketones | Protoporphyrin | C34H34N4O4 | Alkaloid |
| Baimuxinic acid | C15H24O3 | Terpenoids | RA-XXI | C41H50N6O9 | Polypeptide |
| bis(2-ethylhexyl) phthalate | C24H38O4 | Carboxylic acid | Rel-2R-Methoxy-4R-furanogermacr-1(10)E-en-6-one | C16H22O3 | Terpenoids |
| Calycosin-7-O-beta-D-glucopyranoside | C22H22O10 | Flavonoids | Rhapontisterone R1 | C29H42O9 | Steroid |
| cis-Methylisoeugenol | C11H14O2 | Phenols | Salireposide | C20H22O9 | Glycosides |
| Cistachlorin | C9H13ClO3 | Terpenoids | Semiaquilegoside A | C26H38O9 | Terpenoids |
| Cnidilide | C12H18O2 | Heterocyclic compound | SessilifoliAmide B | C17H27NO3 | Alkaloid |
| Crotozambefuran B | C23H28O7 | Terpenoids | Taibairubescensin A | C24H34O7 | Terpenoids |
| Cycloleucine | C6H11NO2 | Amino acid | Thaliporphine | C20H23NO4 | Alkaloid |
| Cynanoside A | C41H62O15 | Glycosides | Torilin | C22H32O5 | Terpenoids |
| Daturametelin A | C34H48O9 | Futo lactone | Trichilinin D | C37H44O8 | Terpenoids |
| Dehydrocrebanine | C20H19NO4 | Alkaloid | Tuberosine A | C19H21NO5 | Alkaloid |
| Delphamine | C24H39NO7 | Alkaloid | Tuberosine B | C10H11NO3 | Alkaloid |
| Didehydrostemofoline | C22H27NO5 | Alkaloid | Tuberostemonine H | C22H33NO4 | Alkaloid |
| Diisobutyl phthalate | C16H22O4 | Carboxylic acid | Valerophenone | C11H14O | Ketones |
| DL-Tyrosine | C9H11NO3 | Amino acid | Zeatin | C10H13N5O | Nucleosides |
| Estriol | C18H24O3 | Steroid | Zingiberone | C11H14O3 | Phenols |

**Table S2 The composition of AEHE**

| **Name** | **Formula** | **Classification** | **Name** | **Formula** | **Classification** |
| --- | --- | --- | --- | --- | --- |
| (-)-Drimenol | C_15_H_26_O | Terpenoids | Galdosol | C_20_H_24_O_5_ | Terpenoids |
| (+)-7,8-Didehydroarctigenin | C_21_H_22_O_6_ | Lignan | Gastrodioside | C_20_H_24_O_8_ | Glycosides |
| (+)-Curcumenol | C_15_H_22_O_2_ | Terpenoids | Gibberellin A119 | C_19_H_24_O_5_ | Terpenoids |
| (2E,4Z,8E)-1-Piperidino-9-(1,3- benzodioxole-5- yl)-2,4,8-nonatriene-1-one | C_21_H_25_NO_3_ | Alkaloid | Gingerenone B | C_22_H_26_O_6_ | Phenols |
| (4e)-1,5-Bis(4-hydroxyphenyl)-2-(hydroxymethyl)-4-penten-1-ol | C_18_H_20_O_4_ | Phenols | Ginsenoside Rk3 | C_36_H_60_O_8_ | Terpenoids |
| (4e)-1,5-Bis(4-hydroxyphenyl)-2-(methoxymethyl)-4-penten-1-ol | C_19_H_22_O_4_ | Phenols | Glabcensin V | C_24_H_34_O_7_ | Terpenoids |
| (5R)-1-(3,4-Dimethoxyphenyl)-5-hydroxydecan-3- one | C_18_H_28_O_4_ | Ketones | Gondoic acid | C_20_H_38_O_2_ | Fatty acid |
| (E,8R)-8-Hydroxyoctadec-9-enoic acid | C_18_H_34_O_3_ | Carboxylic acid | Guaia-1(10),11-dien-9-one | C_15_H_22_O | Ketones |
| (R)-1,5-Dimethylhexylamine | C_8_H_19_N | Amide | Heptadecene | C_17_H_34_ | Phenolphthalein |
| (Z)6,(Z)9-Pentadecadien-1-ol | C_15_H_28_O | Fatty acid | Hosenkoside C | C_48_H_82_O_20_ | Terpenoids |
| 1-(3-Methoxy-4- hydroxyphenyl)-4- undecene-3-one | C_18_H_26_O_3_ | Phenols | Hydroxysesamone | C_15_H_14_O_5_ | Quinones |
| 1-(4-Hydroxybenzyl)-4-methoxy-9,10-dihydrophenanthrene-2,7-diol | C_22_H_20_O_4_ | Aromatic hydrocarbons | Ialibinone E | C_18_H_24_O_4_ | Phenols |
| 1,1,3,3-tetrabutoxy-2-propanone | C_19_H_38_O_5_ | Ketones | Imperialine | C_27_H_43_NO_3_ | Alkaloid |
| 1,2-Benzothiazole | C_7_H_5_NS | Alkaloid | Isodunnianol | C_27_H_26_O_3_ | Terpenoids |
| 1,4-Benzoquinone | C_6_H_4_O_2_ | Quinones | Isoginsenoside-Rh3 | C_36_H_60_O_7_ | Terpenoids |
| 12-Tetradecanoylphorbol-13-acetate | C_36_H_56_O_8_ | Terpenoids | Isoincensole acetate | C_21_H_34_O_3_ | Terpenoids |
| 14-Deoxy-11-oxoandrographolide | C_20_H_28_O_5_ | Terpenoids | Isolariciresinol | C_20_H_24_O_6_ | Lignan |
| 14-Methylhexadecanoic acid | C_17_H_34_O_2_ | Fatty acid | Isomurralonginol acetate | C_17_H_18_O_5_ | Coumarin |
| 1beta- Glucogeniposide | C_17_H_26_O_10_ | Terpenoids | Jasmololone | C_11_H_16_O_2_ | Terpenoids |
| 1-Butoxy-2-ethyl-1-hexene | C_12_H_24_O | Phenolphthalein | Kanshone E | C_15_H_20_O_4_ | Terpenoids |
| 1-Isochromanone | C_9_H_8_O_2_ | Coumarin | Kudzusapogenol A | C_30_H_50_O_5_ | Terpenoids |
| 1-O-(beta-d-Glucopyranosyl)-(2s,3r,4e,8z)-2-[(2r)-2-hydroxyhexadecanoylamino]-4,8-octadecadiene-1,3-diol | C_40_H_75_NO_9_ | Glycolipid | Lappadilactone | C_30_H_38_O_6_ | Terpenoids |
| 2, 6-dimethoxybenzoquinone | C_8_H_8_O_4_ | Quinones | Lauric acid | C_12_H_24_O_2_ | Fatty acid |
| 2,4-Diacetoxypentane | C_9_H_16_O_4_ | Phenolphthalein | Lecithin | C_42_H_81_NO_8_P | Lipid |
| 2,5,9-Trimethylcycloundeca-4,8-dienone | C_14_H_22_O | Ketones | Licoriisoflavan A | C_27_H_34_O_5_ | Flavonoids |
| 21-O-Methyl-5- pregnene-3beta,1 7beta,21-tetrol-20-one | C_22_H_34_O_5_ | Ketones | Linolenyl alcohol | C_18_H_32_O | Fatty alcohol |
| 21-O-Methyltoosendanpentol | C_31_H_52_O_6_ | Terpenoids | L-isoleucine | C_6_H_13_NO_2_ | Amide |
| 27-O-(E)-Coumaroyl-ursolic acid | C_39_H_54_O_6_ | Terpenoids | Lupinifolin | C_25_H_26_O_5_ | Flavonoids |
| 2alpha-Hexadecyloxirane | C_18_H_36_O | Ethers | Machilin D | C_20_H_24_O_5_ | Lignan |
| 2-Hydroxybenzyl-3-hydroxybenzoate | C_14_H_12_O_4_ | Phenolphthalein | Majonoside R2 | C_41_H_70_O_14_ | Terpenoids |
| 2-Icosenal | C_20_H_38_O | Fatty aldehyde | Methoxystemokerrin-N-oxide | C_23_H_33_NO_6_ | Alkaloid |
| 2-Monoolein | C_21_H_40_O_4_ | Glyceride | Methyl 11-eicosenoate | C_21_H_40_O_2_ | Fatty acid |
| 2-Pentadecanone | C_15_H_30_O | Ketones | Methyl 2alpha-methoxyursolate | C_32_H_52_O_4_ | Phenolphthalein |
| 3,4-Dihydroxydihydgaroaroiuran | C_16_H_28_O_2_ | Phenolphthalein | Methyl 4-hydroxycinnamate | C_10_H_10_O_3_ | Carboxylic acid |
| 3,7,7-Trimethylbicyclo[4.1.0]hept-3-ene-2,5-dione | C_10_H_12_O_2_ | Terpenoids | Methyl 6-octadecenoate | C_19_H_36_O_2_ | Phenolphthalein |
| 3-Acetylpomolic acid | C_32_H_50_O_5_ | Terpenoids | Methyl stearate | C_19_H_38_O_2_ | Fatty ester |
| 3-beta-p-Hydroxybenzoyldehydrotumulosic acid | C_38_H_52_O_6_ | Terpenoids | Methylbenzylamin e | C_8_H_11_N | Alkaloid |
| 3-Butyl-4- methylphthalic acid | C_13_H_16_O_4_ | Carboxylic acid | Methylparaben | C_8_H_8_O_3_ | Phenols |
| 3-Hydroxyblancoxanthone | C_23_H_22_O_6_ | Heterocyclic compound | Mussaenosidic acid | C_16_H_24_O_10_ | Terpenoids |
| 3-O-beta-D-Glucopyranosylplatycodigenin | C_36_H_58_O_12_ | Terpenoids | Myristic acid | C_14_H_28_O_2_ | Fatty acid |
| 3-Octadecenoic acid | C_18_H_34_O_2_ | Fatty acid | Myristicanol A | C_23_H_30_O_8_ | Phenolphthalein |
| 4-Epiisocembrol | C_20_H_34_O | Terpenoids | Neotuberostemonol | C_22_H_31_NO_5_ | Alkaloid |
| 4'-Methylcapillarisin | C_17_H_14_O_6_ | Heterocyclic compound | N-Isobutyl-(2E,4E)-dodecadienAmide | C_16_H_29_NO | Fatty amide |
| 4-tert-Butyl-2-methylphenol | C_11_H_16_O | Phenols | N-Isobutyldeca-trans-2-trans-4-dienAmide | C_14_H_25_NO | Fatty amide |
| 5,7,4'-Trimethylapigenin | C_18_H_16_O_5_ | Flavonoids | Nobilin | C_20_H_26_O_5_ | Terpenoids |
| 5alpha-Hydroxytriptonide | C_20_H_22_O_7_ | Terpenoids | Octacosanedioic acid | C_28_H_54_O_4_ | Carboxylic acid |
| 5-Methoxy dehydrodiisoeugenol | C_21_H_24_O_5_ | Phenols | Octadecadienoic acid | C_18_H_32_O_2_ | Fatty acid |
| 7-Methoxyrosmanol | C_21_H_28_O_5_ | Terpenoids | Octadecanoic acid, 8-quinolinyl ester | C_27_H_41_NO_2_ | Alkaloid |
| 8-Octadecenoic acid | C_18_H_34_O_2_ | Fatty acid | OleAmide | C_18_H_35_NO | Fatty amide |
| 9,10-Dihydroxystearic acid | C_18_H_36_O_4_ | Fatty acid | Orotinin | C_25_H_26_O_6_ | Flavonoids |
| 9-Hexadecenoic acid | C_16_H_30_O_2_ | Fatty acid | Oxypeucedanin | C_16_H_14_O_5_ | Coumarin |
| 9-Methyl-3-undecene | C_12_H_24_ | Fatty acid | Palmitic acid | C_16_H_32_O_2_ | Fatty acid |
| Abrisapogenol C | C_30_H_50_O_4_ | Saponin | Palmitic acid ethyl ester | C_18_H_36_O_2_ | Fatty acid |
| Abrisapogenol I | C_30_H_46_O_5_ | Saponin | Palmitic acid N-butyl ester | C_20_H_40_O_2_ | Fatty acid |
| Acenaphthylene | C_12_H_8_ | Aromatic hydrocarbons | Pariposide D | C_39_H_60_O_15_ | Steroid |
| Acortatarin A | C_12_H_15_NO_5_ | Alkaloid | pedunculoside | C_36_H_58_O_10_ | Terpenoids |
| Adenosine | C_10_H_13_N_5_O_4_ | Nucleosides | Pentadecanoic acid | C_15_H_30_O_2_ | Fatty acid |
| Allixin | C_12_H_18_O_4_ | Heterocyclic compound | Phanginin D | C_22_H_30_O_5_ | Terpenoids |
| Apioglycyrrhizin | C_41_H_62_O_14_ | Terpenoids | Phanginin G | C_21_H_28_O_5_ | Terpenoids |
| Arctigenin | C_21_H_24_O_6_ | Lignan | Phanginin H | C_21_H_28_O_4_ | Terpenoids |
| Artemisyl acetate | C_12_H_20_O_2_ | Terpenoids | Phanginin J | C_21_H_26_O_5_ | Terpenoids |
| Aryl-8-oxa-bicyclo [3,2,1]-oct-3-en-2-one | C_16_H_18_O_6_ | Ketones | Phthalic anhydride | C_8_H_4_O_3_ | Carboxylic acid |
| Benzofuran | C_8_H_6_O | Heterocyclic compound | Pipercide | C_22_H_29_NO_3_ | Alkaloid |
| bis(2-ethylhexyl) phthalate | C_24_H_38_O_4_ | Carboxylic acid | Pisatin | C_17_H_14_O_6_ | Flavonoids |
| Blumenol B | C_13_H_22_O_3_ | Terpenoids | Polyanthinin | C_26_H_32_O_5_ | Coumarin |
| Borreriagenin | C_10_H_14_O_5_ | Terpenoids | Polygonatoside D | C_45_H_72_O_18_ | Steroid |
| Bupleuroside XI | C_48_H_80_O_16_ | Terpenoids | Pratensein | C_16_H_12_O_6_ | Flavonoids |
| Butein | C_15_H_12_O_5_ | Ketones | Protoporphyrin | C_34_H_34_N_4_O_4_ | Alkaloid |
| Calaxin | C_19_H_20_O_6_ | Terpenoids | Protostemotinine | C_23_H_29_NO_6_ | Alkaloid |
| Calenduloside E Methyl Ester | C_37_H_58_O_9_ | Terpenoids | Prunin | C_21_H_22_O_10_ | Flavonoids |
| Calycosin-7-O- beta-D- glucopyranoside | C_22_H_22_O_10_ | Flavonoids | Pseudoginsenoside Rp1 | C_41_H_64_O_13_ | Saponin |
| Campesteryl ferulate | C_38_H_56_O_4_ | Steroid | Pseudolaric acid D | C_20_H_30_O_3_ | Terpenoids |
| Cantoniensistriol | C_30_H_50_O_3_ | Terpenoids | Ramalic acid | C_18_H_18_O_7_ | Phenols |
| Chasmanine | C_25_H_41_NO_6_ | Alkaloid | Rel-2R-Methoxy-4R-furanogermacr-1(10)E-en-6-one | C_16_H_22_O_3_ | Terpenoids |
| Chimyl alcohol | C_19_H_40_O_3_ | Fatty acid | Salireposide | C_20_H_22_O_9_ | Glycosides |
| Chiratone | C_30_H_50_O_2_ | Terpenoids | Sanleng acid | C_18_H_34_O_5_ | Organic acid |
| Chuanbeinone | C_27_H_43_NO_2_ | Alkaloid | Schisandrol A | C_24_H_32_O_7_ | Lignan |
| cis-Methylisoeugenol | C_11_H_14_O_2_ | Phenols | Schisanlactone E | C_30_H_44_O_4_ | Terpenoids |
| Citronellal | C_10_H_18_O | Terpenoids | Sesibiricin | C_20_H_24_O_4_ | Coumarin |
| Cnidilide | C_12_H_18_O_2_ | Heterocyclic compound | SessilifoliAmide B | C_17_H_27_NO_3_ | Alkaloid |
| Columbianetin | C_14_H_14_O_4_ | Coumarin | Skimmetin | C_9_H_6_O_3_ | Coumarin |
| Curcarabranol B | C_15_H_24_O_3_ | Terpenoids | Skimmianine | C_14_H_13_NO_4_ | Alkaloid |
| Curculigosaponin L | C_42_H_72_O_13_ | Terpenoids | Solanone | C_13_H_22_O | Terpenoids |
| Cyclamigenin B | C_30_H4_6_O_4_ | Terpenoids | Suspenolic acid | C_10_H_14_O_4_ | Phenols |
| Cycloleucine | C_6_H_11_NO_2_ | Amide | Tenacissimoside B | C_44_H_62_O_14_ | Terpenoids |
| Daturametelin A | C_34_H_48_O_9_ | Futo lactone | Tenasogenin | C_26_H_40_O_6_ | Steroid |
| Dehydroabietic acid methyl ester | C_21_H_30_O_2_ | Resin acid | Tetradecanal | C_14_H_28_O | Fatty aldehyde |
| Dehydroevodiamine | C_19_H_15_N_3_O | Alkaloid | TetradecanAmide | C_14_H_29_NO | Fatty amide |
| Dehydropachymic acid | C_33_H_50_O_5_ | Terpenoids | Tetradecenoic acid A | C_14_H_26_O_2_ | Fatty acid |
| Delphamine | C_24_H_39_NO_7_ | Alkaloid | Timosaponin O | C_46_H_78_O_20_ | Saponin |
| Descurainolide A | C_12_H_14_O_5_ | Lactone | Tomentogenin | C_21_H_36_O_5_ | Steroid |
| Dibutyl phthalate | C_16_H_22_O_4_ | Carboxylic acid | Torilin | C_22_H_32_O_5_ | Terpenoids |
| Dictamdiol | C_15_H_18_O_5_ | Terpenoids | Trichilinin D | C_37_H_44_O_8_ | Terpenoids |
| Didehydrostemofoline | C_22_H_27_NO_5_ | Alkaloid | Tuberosine B | C_10_H_11_NO_3_ | Alkaloid |
| Dihydro-4,4-dimethyl-2,3-Furandione | C_6_H_8_O_3_ | Lactone | Tuberostemonine H | C_22_H_33_NO_4_ | Alkaloid |
| Dihydro-beta-ionol | C_13_H_24_O | Terpenoids | Turpinionosides D | C_19_H_34_O_8_ | Terpenoids |
| Diisooctyl succinate | C_20_H_38_O_4_ | Phenolphthalein | Ursolic acid | C_30_H_48_O_3_ | Terpenoids |
| Dimethyl camphorate | C_12_H_20_O_4_ | Carboxylic acid | Valerophenone | C_11_H_14_O | Ketones |
| Dioscin | C_45_H_72_O_16_ | Steroid | Vilmorrianine C | C_35_H_49_NO_9_ | Alkaloid |
| DL-Tyrosine | C_9_H_11_NO_3_ | Amide | Vina-ginsenoside R1 | C_44_H_74_O_15_ | Terpenoids |
| Docosanoic acid | C_22_H_44_O_2_ | Fatty acid | Virgatusin | C_23_H_28_O_7_ | Lignan |
| Docosene | C_22_H_44_ | Fatty acid | Wulingzhic acid A | C_20_H_32_O_5_ | Terpenoids |
| Eleutheroside A | C_35_H_60_O_6_ | Steroid | Z-6-Hydroxy-7-Methoxydihydroligustilide | C_13_H_18_O_4_ | Phenolphthalein |
| ent-Kauran-16α,17- diol | C_20_H_34_O_2_ | Terpenoids | Zeatin | C_10_H_13_N_5_O | Nucleosides |
| Estriol | C_18_H_24_O_3_ | Steroid | Zederone | C_15_H_18_O_3_ | Terpenoids |
| Ethyl 3-(2-hydroxyphenyl)acrylate | C_11_H_12_O_3_ | Phenols | Zedoalactone B | C_15_H_20_O_5_ | Terpenoids |
| Feroxin A | C_17_H_24_O_8_ | Phenolphthalein | Zingiberone | C_11_H_14_O_3_ | Phenols |
| Galdosol | C_20_H_24_O_5_ | Terpenoids | Ziyu glycoside Ⅱ | C_35_H_56_O_8_ | Saponin |

**Table S3 Names of DEPs in the WE-H group**

| **Protein id** | **Protein Name** |
| --- | --- |
| Q4KM66 | LOC500183 protein |
| A6JII4 | Progastricsin (Pepsinogen C) |
| P08932 | T-kininogen 2 |
| A6J5U7 | Fibrinogen, gamma polypeptide, isoform CRA_a |
| F7FJ08 | Vitamin D-binding protein |
| H6X2W7 | Pentraxin family member |
| A6KH80 | Mast cell peptidase 2 |
| Q9WUW9 | Sulfotransferase 1C2A |
| P20762 | Ig gamma-2C chain C region |
| Q99PS8 | Histidine-rich glycoprotein |
| F7FC04 | Beta-2-glycoprotein 1 |
| G3V843 | Prothrombin |
| A0A8I6ABI1 | Serpin family G member 1 |
| A0A8I5Y6S4 | Kininogen 2 |
| A0A8I6A7H1 | Deoxyribonuclease |
| P25809 | Creatine kinase U-type, mitochondrial |
| A6KB24 | WD repeat domain 27 (Predicted) (Fragment) |
| A0A8I6GLM4 | Lymphocyte-specific protein 1 |
| A6KKT6 | Catechol-O-methyltransferase domain containing 1 (Predicted), isoform CRA_b |
| F1LPR6 | Immunoglobulin heavy constant epsilon |
| A6ITJ0 | Phospholipase A2 |
| A0A8I6ACZ6 | Complement C8 alpha chain |
| A6HJL1 | RCG33119 |
| A6K3Q4 | CTTNBP2 N-terminal like (Predicted), isoform CRA_b |
| F7FQ32 | RCG59057, isoform CRA_a |
| A6IWK1 | Protein Z, vitamin K-dependent plasma glycoprotein (Predicted), isoform CRA_a |
| A0A8I6A189 | Fas associated factor 1 |
| A6KB27 | RCG22919, isoform CRA_a |
| D3Z9I1 | Cytochrome c oxidase assembly factor 3 |
| G3V729 | Proteoglycan 2 |
| M0R7B4 | H1.3 linker histone, cluster member |
| A6JCA2 | RCG24811, isoform CRA_b |
| Q923Z2 | Tropomyosin 1, alpha, isoform CRA_a |
| A6KUU3 | RCG43755 |
| A0A8I6AA89 | Uncharacterized protein |
| F1LPN4 | Presenilin associated, rhomboid-like |
| A0A8I6A7N3 | Cullin 5 |
| D3ZCD6 | Ig-like domain-containing protein |
| A6JIA9 | Similar to RIKEN cDNA 1810055E12 (Predicted), isoform CRA_b |
| A0A8I6AEN6 | Fc receptor-like 1 |
| A6JQJ5 | Secreted phosphoprotein 24 |
| A6JN47 | RCG31649, isoform CRA_b |
| A0A8I6AH54 | Hydroxymethylglutaryl-CoA synthase |
| D3ZZZ0 | AT hook containing transcription factor 1 |
| A0A8I5ZRM7 | Death-associated protein |
| D3ZFS7 | palmitoyl-protein hydrolase |
| Q63356 | Unconventional myosin-Ie |
| A0A8L2QTR5 | Mucin like 3 |
| A0A8I6AKA2 | RNA-binding protein with serine-rich domain 1 |
| A0A0G2K278 | Glutathione peroxidase |
| A6IPP7 | Small muscular protein |
| Q09325 | Alpha-1,3-mannosyl-glycoprotein 2-beta-N-acetylglucosaminyltransferase |
| A6HXN5 | Plakophilin 3 (Predicted), isoform CRA_e |
| A6KMB6 | Oxysterol-binding protein |
| A0A8I5ZLL3 | Rho/Rac guanine nucleotide exchange factor 18 |
| A0A8I5ZRY8 | Papilin, proteoglycan-like sulfated glycoprotein |
| A0A8I5Y7F9 | Tyrosine-protein phosphatase non-receptor type |
| Q78EJ9 | Calpain-8 |
| A0A8I6AAW5 | Alpha-(1,6)-fucosyltransferase |
| A0A8L2QLK6 | Histone H2A |
| D3ZFP4 | DNA replication licensing factor MCM3 |
| A6KHA0 | Poly [ADP-ribose] polymerase |
| P70705 | Copper-transporting ATPase 1 |
| M0RD88 | Sperm motility kinase 2A |
| A0A8I6A4W6 | Adenine nucleotide translocase lysine methyltransferase |
| D3ZBP4 | [F-actin]-monooxygenase MICAL1 |
| A6K1L0 | ER lumen protein-retaining receptor |
| A0A8I5ZP38 | Syntaxin 8 |
| A6J2Q0 | Uncharacterized protein RGD1304762 |
| A0A8I6A4B8 | ADAM metallopeptidase domain 28 |
| F1LXF5 | Guided entry of tail-anchored proteins factor 4 |
| M0R5N4 | Prefoldin subunit 4 |
| A6JGC3 | RCG20491, isoform CRA_a |
| A0A8I6AZ21 | DENN domain containing 2D |
| A6HVH4 | Fatty acid-binding protein, intestinal |

**Table S4 Names of DEPs in the AE-H group**

| **Protein id** | **Protein Name** |
| --- | --- |
| A6IH94 | Zinc finger homeodomain 4 (Predicted) |
| Q5U329 | Anion exchange protein |
| A6JM53 | RCG55866 |
| A0A140UHX6 | Spectrin beta chain |
| G3V843 | Prothrombin |
| F1LTN6 | Ig-like domain-containing protein |
| Q68FT8 | RCG33981, isoform CRA_a |
| A0A8I6G388 | Embigin |
| A6IE20 | Caveolin |
| A6KGR9 | Dolichyl-diphosphooligosaccharide--protein glycosyltransferase subunit DAD1 |
| A6HE76 | SAR1 gene homolog B (S. cerevisiae), isoform CRA_b |
| Q62812 | Myosin-9 |
| A6J6C0 | Farnesyl pyrophosphate synthase |
| A0A8I5ZRM7 | Death-associated protein |
| B5DF55 | Signal transducing adaptor molecule |
| A6HIX0 | RCG34563, isoform CRA_c |
| Q5BK10 | Calpain-13 |
| A6IAC6 | Capping protein (Actin filament), gelsolin-like, isoform CRA_a |
| D4A573 | Small integral membrane protein 15 |
| A6HWS5 | Zinc finger Ran-binding domain-containing protein 2 |
| F7EY20 | Chemokine (C-X-C motif) ligand 7, isoform CRA_b |
| A0A096MJN6 | PX domain-containing protein |
| G3V7Y7 | Sodium/hydrogen exchanger |
| Q62847 | Gamma-adducin |
| A6KTU9 | Apolipoprotein M |
| Q5PQQ1 | tRNA modification GTPase GTPBP3, mitochondrial |
| A6ITQ9 | Ciliary rootlet coiled-coil, rootletin (Predicted) |
| A0A8I6GFD5 | procollagen-lysine 5-dioxygenase |
| D3ZCD6 | Ig-like domain-containing protein |
| D3ZFS7 | palmitoyl-protein hydrolase |
| A0A8L2UIL5 | Programmed cell death 10 |
| A6JS84 | RCG36803, isoform CRA_b |
| Q6AZ40 | Prpsap2 protein |
| A0A8I6AJ69 | Dipeptidylpeptidase 7 |
| A6HXN5 | Plakophilin 3 (Predicted), isoform CRA_e |
| Q78EJ9 | Calpain-8 |
| Q4V797 | Interferon-gamma-inducible GTPase Ifgga1 protein |
| A0A8I6AM36 | Ubiquitin-conjugating enzyme E2H |
| A6JDH7 | Similar to expressed sequence AA536743 (Predicted) |
| P70705 | Copper-transporting ATPase 1 |
| G3V645 | 2-5-oligoadenylate synthase-like protein 1 |
| A6KSQ7 | RCG39258, isoform CRA_c |
| D3ZTE2 | VPS50 subunit of EARP/GARPII complex |
| A0A8I5ZPF4 | D-ribitol-5-phosphate cytidylyltransferase |
| A6IA46 | Similar to Pleckstrin homology domain-containing protein family A member 1 (Tandem PH domain containing protein-1) (Predicted), isoform CRA_b |
| Q497A6 | Brd4 protein (Fragment) |
| Q9Z2G3 | ATP-citrate (Pro-S-)-lyase (Fragment) |
| A6J2A2 | RCG21555, isoform CRA_b |
| A0A8I5ZXI9 | X-ray repair cross complementing 6 |
